# Tumor necrosis factor alpha is a promising circulating biomarker for the development of obstructive sleep apnea syndrome: a meta-analysis

**DOI:** 10.18632/oncotarget.15203

**Published:** 2017-02-08

**Authors:** Qingsheng Li, Xin Zheng

**Affiliations:** ^1^ Department of Emergency Pediatrics, The First Affiliated Hospital of Fujian Medical University, Fuzhou, China; ^2^ Department of Basic Medicine, Fujian Health Collage, Fuzhou, China

**Keywords:** obstructive sleep apnea syndrome, tumor necrosis factor alpha, meta-analysis, mean difference

## Abstract

Obstructive sleep apnea syndrome (OSAS) is a chronic inflammatory disorder. The relationship between tumor necrosis factor alpha (TNF-alpha) and OSAS has been widely evaluated, but the results thus far remain inconclusive. We thereby decided to quantify the changes of TNF-alpha between OSAS patients and controls by a meta-analysis. This study complies with the MOOSE guidelines. Two reviewers independently searched articles and abstracted relevant data. In total, 47 articles (59 studies) were analyzed, including 2857 OSAS patients and 2115 controls. Overall, OSAS patients had a significantly higher level of circulating TNF-alpha than controls (weighted mean difference [WMD]: 9.66 pg/mL, 95% confidence interval [CI]: 8.66 to 11.24, *P*<0.001), but with significant heterogeneity (*I*^2^: 99.7%). After adjusting for potential missing studies, the overall estimate was weakened but still significant (filled WMD: 2.63 pg/mL, 95% CI: 2.56 to 2.70, *P*<0.001). When studies were stratified by OSAS severity, the changes in circulating TNF-alpha between patients and controls increased gradually with the more severe grades of OSAS. In patients with mild, mild-to-moderate, moderate, moderate-to-severe and severe OSAS, circulating TNF-alpha was higher than respective controls by 0.99, 1.48. 7.79, 10.08 and 8.85 pg/mL, with significant heterogeneity (*I*^2^: 91.2%, 74.5%, 97.6%, 99.0% and 98.1%). In conclusion, our findings demonstrated that circulating TNF-alpha was significantly higher in OSAS patients than in controls, and this difference became more pronounced with the more severe grades of OSAS, indicating that TNF-alpha might be a promising circulating biomarker for the development of OSAS.

## INTRODUCTION

Obstructive sleep apnea syndrome (OSAS) is a chronic inflammatory disorder featured by recurrent bouts of partial or complete upper airway obstruction during sleeping [1]. OSAS poses a major burden on individual and public health, as it respectively affects 10% and 17% of middle-aged (30-49 years old) and aged (50-70 years old) men, and 3% and 9% of middle-aged and aged women [2]. It is worth noting that affected individuals are more likely to suffer cardio- and cerebro-vascular diseases, such as hypertension, heart failure and stroke [3, 4]. At present, continuous positive airway pressure (CPAP) ranks as the main treatment option for patients with moderate or severe OSAS, and it can assist in reducing systematic inflammation in the airways of OSAS patients [5]. Hence, understanding the inflammation process may offer a possible clue to understanding the molecular mechanisms behind the pathogenesis of OSAS.

Several lines of evidence from animal experiments and clinical investigations have indicated that the presence of OSAS is associated with the increased production of inflammatory mediators [6, 7]. Tumor necrosis factor alpha (TNF-alpha) is a key modulator of systematic inflammation [8–10], and TNF inhibition has proven to ameliorate the progression of OSAS [11]. Moreover, some researchers have observed a significant high level of circulating TNF-alpha in OSAS patients vis-à-vis healthy individuals [12–18], whereas others did not [19, 20]. The probable causes are multifaceted, relating to statistical power, research design, genetic heterogeneity or dietary habit. The inconsistent reported relations and many resulting debates motivated us to postulate that circulating TNF-alpha might be a promising intermediate biomarker for predicting OSAS development. To uphold this postulation, we conducted an extensive search of current literature for published articles that reported data on circulating TNF-alpha between OSAS patients and controls, and thereby quantified the changes of TNF-alpha by a meta-analysis.

## RESULTS

After searching three public databases, a total of 171 articles written in English language were indexed. After reviewing the title and abstract of each article, 99 were excluded for definitive reasons. After reading the full text of the rest 72 potential articles, 25 were further excluded, leaving 47 qualified articles in this meta-analysis according to the preset inclusive criteria [12–58]. Because 9 articles provided data by OSAS severity, 1 article by hypertension and 1 article by obesity, there were a total of 59 independent studies involving 2857 OSAS patients and 2115 controls in the final analysis. The baseline characteristics of 59 studies are summarized in Table 1 and Supplementary Table 1.

**Table 1:** The baseline characteristics of 59 studies in the present meta-analysis

First author	Year	Country	OSAS severity	Type	Sample size		Age (years)		Male gender		BMI (kg/m2)		Hypertension		Diabetes		AHI (events/h)		TNF-alpha (pg/mL)	
					Pati's	Cont's	Pati's	Cont's	Pati's	Cont's	Pati's	Cont's	Pati's	Cont's	Pati's	Cont's	Pati's	Cont's	Pati's	Cont's
Vgontzas AN	1997	USA	All	C.S.	12	10	40.9	24.1	0.92	1.00	40.5	24.6	N.R.	N.R.	N.R.	N.R.	63.7	0.0	2.51	1.17
Liu H	2000	China	All	C.S.	22	16	47.4	47.6	0.68	0.69	27.6	23.1	N.R.	N.R.	N.R.	N.R.	44.0	4.3	299.09	101.88
Teramoto S	2003	Japan	All	C.S.	40	40	N.R.	N.R.	0.85	N.R.	N.R.	N.R.	0.00	0.00	0.00	0.00	N.R.	N.R.	9.50	4.40
Alberti A	2003	Italy	Moderate-to-severe	C.S.	18	20	52.7	51.3	0.72	0.70	26.5	22.1	0.33	0.00	0.00	0.00	18.2	N.R.	9.70	6.30
Minoguchi K (a)	2004	Japan	Mild	C.S.	12	12	51.0	47.5	1.00	1.00	26.1	22.3	0.08	0.00	0.08	0.00	9.0	2.1	1.80	1.12
Minoguchi K (b)	2004	Japan	Moderate	C.S.	12	12	49.2	47.5	1.00	1.00	29.1	22.3	0.25	0.00	0.08	0.00	59.2	2.1	2.34	1.12
Imagawa S	2004	Japan	Severe	C.S.	110	45	N.R.	N.R.	N.R.	N.R.	28.5	22.9	0.00	0.00	0.00	0.00	N.R.	N.R.	28.60	25.00
Ciftci TU	2004	Turkey	All	C.S.	43	22	49.6	47.2	1.00	1.00	31.9	31.0	0.00	0.00	0.00	0.00	33.2	1.6	4.60	3.29
Tam CS	2006	Australia	All	C.S.	44	69	7.3	7.6	0.68	0.64	19.4	17.9	0.00	0.00	0.00	0.00	N.R.	N.R.	5.30	4.70
Ryan S (a)	2006	Ireland	Mild-to-moderate	N.S.	35	30	42.0	41.0	1.00	1.00	32.9	30.7	0.00	0.00	0.00	0.00	15.9	1.2	4.15	3.21
Ryan S (b)	2006	Ireland	Severe	N.S.	31	30	43.0	41.0	1.00	1.00	32.1	30.7	0.00	0.00	0.00	0.00	56.6	1.2	6.19	3.21
Kobayashi K	2006	Japan	Severe	C.S.	35	16	51.4	41.0	0.86	0.81	27.9	27.4	0.49	0.44	0.20	0.19	52.3	9.0	1.11	0.62
Bravo Mde L	2007	Spain	Moderate-to-severe	C.S.	50	20	52.3	47.4	1.00	1.00	30.9	28.4	0.68	0.00	0.00	0.00	48.9	2.5	0.89	0.42
Li Y	2008	China	All	N.S.	68	22	48.3	43.0	0.74	0.64	25.7	23.3	0.00	0.00	0.00	0.00	31.4	2.9	113.80	87.30
Li AM	2008	China	All	C.S.	47	95	11.1	10.7	0.70	0.67	N.R.	N.R.	N.R.	N.R.	N.R.	N.R.	14.1	0.7	0.40	0.50
Kanbay A	2008	Turkey	All	C.S.	106	32	51.4	44.8	0.58	0.59	31.1	28.3	0.47	0.38	0.24	0.17	40.1	2.0	114.15	34.25
Constantinidis J (a)	2008	Greece	All	C.S.	13	12	45.1	N.R.	1.00	1.00	33.4	34.9	N.R.	N.R.	N.R.	N.R.	23.6	3.4	124.64	78.80
Constantinidis J (b)	2008	Greece	All	C.S.	11	15	45.1	N.R.	1.00	1.00	26.1	27.4	N.R.	N.R.	N.R.	N.R.	22.9	3.6	105.00	48.50
Arias MA	2008	Spain	Moderate-to-severe	N.S.	30	15	52.0	48.0	1.00	1.00	30.5	28.7	0.00	0.00	0.00	0.00	43.8	3.7	18.50	11.40
Antonopoulou S	2008	Greece	Moderate-to-severe	C.S.	45	25	52.0	51.0	0.82	0.72	33.5	31.0	0.00	0.00	0.00	0.00	39.0	N.R.	1.40	0.64
Thomopoulos C	2009	Greece	All	C.S.	62	70	48.1	48.1	0.79	0.80	31.9	32.1	1.00	1.00	0.00	0.00	31.6	0.4	2.14	1.26
Tamaki S (a)	2009	Japan	Mild-to-moderate	C.S.	13	13	56.1	35.5	0.85	0.92	24.6	23.6	0.00	0.00	0.00	0.00	18.3	3.8	22.70	17.30
Tamaki S (b)	2009	Japan	Severe	C.S.	20	13	50.5	35.5	0.95	0.92	30.7	23.6	0.00	0.00	0.00	0.00	60.4	3.8	30.20	17.30
Li Y (a)	2009	China	Mild	C.S.	22	22	48.0	43.0	0.68	0.64	25.7	23.3	0.00	0.00	0.00	0.00	14.1	2.9	102.30	87.30
Li Y (b)	2009	China	Moderate	C.S.	22	22	44.0	43.0	0.82	0.64	28.8	23.3	0.00	0.00	0.00	0.00	29.7	2.9	125.00	87.30
Li Y (c)	2009	China	Severe	C.S.	24	22	44.0	43.0	0.71	0.64	28.7	23.3	0.00	0.00	0.00	0.00	70.1	2.9	132.10	87.30
Carneiro G	2009	Brazil	All	C.S.	16	13	40.1	38.8	1.00	1.00	46.9	42.8	0.54	0.69	0.00	0.00	65.7	3.2	10.70	7.50
Bhushan B	2009	India	Moderate-to-severe	C.S.	104	103	46.2	44.0	0.81	0.63	31.5	30.9	0.00	0.00	0.00	0.00	N.R.	N.R.	113.04	76.23
Steiropoulos P	2010	Greece	Moderate	C.S.	38	23	45.5	43.7	0.87	0.74	36.4	34.5	0.00	0.00	0.00	0.00	61.0	5.3	6.72	3.94
Sahlman J	2010	Finland	Mild	C.S.	84	40	50.4	45.6	0.76	0.63	32.5	31.5	0.37	0.33	0.08	0.05	9.6	1.9	1.54	1.17
Li NF (a)	2010	China	Moderate-to-severe	C.S.	113	97	45.5	44.2	0.75	0.76	27.8	26.9	0.00	0.00	0.00	0.00	N.R.	N.R.	19.98	13.10
Li NF (b)	2010	China	Moderate-to-severe	C.S.	134	73	46.1	46.0	0.75	0.74	28.9	27.7	1.00	1.00	0.00	0.00	N.R.	N.R.	22.85	17.32
Kim J (a)	2010	Korea	Moderate	C.S.	9	22	38.0	26.0	N.R.	N.R.	24.4	23.9	0.00	0.00	0.00	0.00	14.4	1.3	14.56	14.40
Kim J (b)	2010	Korea	Severe	C.S.	28	22	42.0	26.0	N.R.	N.R.	28.7	23.9	0.00	0.00	0.00	0.00	52.7	1.3	15.32	14.40
Khalyfa A	2011	USA	All	C.S.	60	80	7.2	7.2	0.50	0.50	N.R.	N.R.	0.00	0.00	0.00	0.00	8.9	0.5	459.80	295.60
Qian X	2012	China	Severe	C.S.	30	40	45.0	46.3	1.00	1.00	29.4	24.1	0.00	0.00	0.03	0.03	N.R.	N.R.	115.00	114.00
Mederios CA (a)	2012	Brazil	Mild-to-moderate	C.S.	15	15	62.6	62.5	0.73	0.40	24.5	25.8	0.73	0.40	0.13	0.07	N.R.	N.R.	0.84	0.32
Mederios CA (b)	2012	Brazil	Severe	C.S.	35	15	65.0	62.5	0.57	0.40	25.9	25.8	0.86	0.40	0.26	0.07	N.R.	N.R.	2.09	0.32
Deboer MD	2012	USA	All	C.S.	9	15	14.2	14.6	0.44	0.67	N.R.	N.R.	0.00	0.00	0.00	0.00	13.5	0.8	0.99	0.98
Fornadi K	2012	German	All	C.S.	25	75	54.0	50.0	0.80	0.49	29.0	26.0	N.R.	N.R.	N.R.	N.R.	N.R.	N.R.	2.20	1.90
Yang D	2013	China	All	C.S.	25	25	54.0	53.0	0.92	0.92	27.4	26.3	0.64	N.R.	0.20	N.R.	24.0	3.0	12.55	5.12
Hargens T	2013	USA	All	C.S.	12	15	22.8	21.1	1.00	1.00	32.4	22.2	0.00	0.00	0.00	0.00	25.4	2.0	950	860
Driessen C	2013	Netherland	All	N.S.	23	25	9.8	12.0	0.43	0.56	21.3	20.0	N.R.	N.R.	N.R.	N.R.	3.6	0.4	15.10	12.30
Doufas AG	2013	USA	All	C.S.	33	15	34.0	31.0	1.00	1.00	26.0	24.0	0.00	0.00	0.00	0.00	13.0	2.4	7.88	7.77
Chen PC (a)	2013	China	Mild	C.S.	23	20	40.0	42.0	0.74	0.75	27.5	26.0	0.00	0.00	0.00	0.00	8.6	3.3	2.80	1.20
Chen PC (b)	2013	China	Moderate	C.S.	21	20	45.0	42.0	0.76	0.75	26.7	26.0	0.00	0.00	0.00	0.00	21.1	3.3	3.80	1.20
Alexopoulos EI (a)	2013	Greece	Mild	C.S.	22	22	6.0	6.8	0.36	0.45	N.R.	N.R.	0.00	0.00	0.00	0.00	2.1	0.5	0.65	0.63
Alexopoulos EI (b)	2013	Greece	Moderate-to-severe	C.S.	24	22	5.7	6.8	0.46	0.45	N.R.	N.R.	0.00	0.00	0.00	0.00	11.5	0.5	0.63	0.63
Yadav R	2014	UK	Moderate-to-severe	C.S.	20	21	49.0	45.0	0.15	0.20	52.0	50.0	0.65	0.50	0.30	0.30	21.3	4.3	87.20	15.50
Nobili V	2014	Italy	All	N.S.	39	26	11.8	11.6	0.56	0.62	28.3	26.4	0.13	0.19	0.03	0.04	4.4	0.5	2.20	6.80
Ciccone M (a)	2014	Italy	Mild	C.S.	26	40	53.7	52.3	0.88	0.85	28.1	28.2	0.00	0.00	0.00	0.00	10.6	2.1	14.42	12.53
Ciccone M (b)	2014	Italy	Moderate-to-severe	C.S.	54	40	52.3	52.3	0.83	0.85	28.8	28.2	0.00	0.00	0.00	0.00	45.1	2.1	22.83	12.53
Zhang Y	2015	China	Moderate-to-severe	C.S.	408	394	48.5	48.8	0.84	0.82	28.8	23.5	0.00	0.00	0.00	0.00	N.R.	N.R.	64.72	30.56
Thunstrom E	2015	Sweden	Moderate-to-severe	N.S.	234	95	65.3	61.4	0.87	0.75	26.8	25.2	0.59	0.45	0.15	0.13	28.9	3.1	5.00	4.20
Leon-Cabrera S	2015	Mexico	Moderate-to-severe	C.S.	29	10	37.2	43.4	0.14	0.80	45.2	23.6	0.00	0.00	0.00	0.00	51.4	7.5	337.90	270.20
Jiang H	2015	China	All	C.S.	135	94	48.7	47.2	0.59	0.59	27.5	27.5	0.00	0.00	0.00	0.00	24.6	1.6	765.77	232.24
De Santis S	2015	Italy	All	C.S.	26	24	41.8	43.7	0.65	0.67	33.0	30.8	0.00	0.00	0.00	0.00	26.2	1.7	122.20	80.20
Lin CC	2016	China	All	N.S.	35	20	46.0	43.0	0.86	0.90	29.2	28.2	0.00	0.00	0.00	0.00	59.3	3.6	25.00	14.00
Ifergane G	2016	Israel	Moderate-to-severe	C.S.	21	22	66.0	66.1	0.38	0.23	29.6	26.8	0.76	0.59	0.24	0.27	N.R.	N.R.	6.39	3.57

Abbreviations: Pati's, patients; Cont's, controls; C.S., cross-sectional case-control study; N.S., nested case-control study; BMI, body mass index; AHI, apnea-hypopnea index; TNF-alpha, tumor necrosis factor alpha; N.R., data not reported.

Of 59 qualified studies, 25 were from Asian countries, 21 from European countries, 5 from North American countries, 3 from South American countries, 3 from cross-continent countries, 1 respectively from Australia and Latin America. 13 studies involved only male individuals, and 7 studies involved underage individuals. Age was reportedly matched between patients and controls by 23 studies, and there were 35 studies involving individuals free of hypertension and diabetes mellitus. There were 51 and 8 cross-sectional and nested case-control studies, respectively. OSAS was diagnosed by polysomnography by 51 studies. As for OSAS severity, mild OSAS was reported by 6 studies, mild-to-moderate OSAS by 3 studies, moderate OSAS by 5 studies, moderate-to-severe OSAS by 14 studies and severe OSAS by 8 studies.

**Figure 1:**
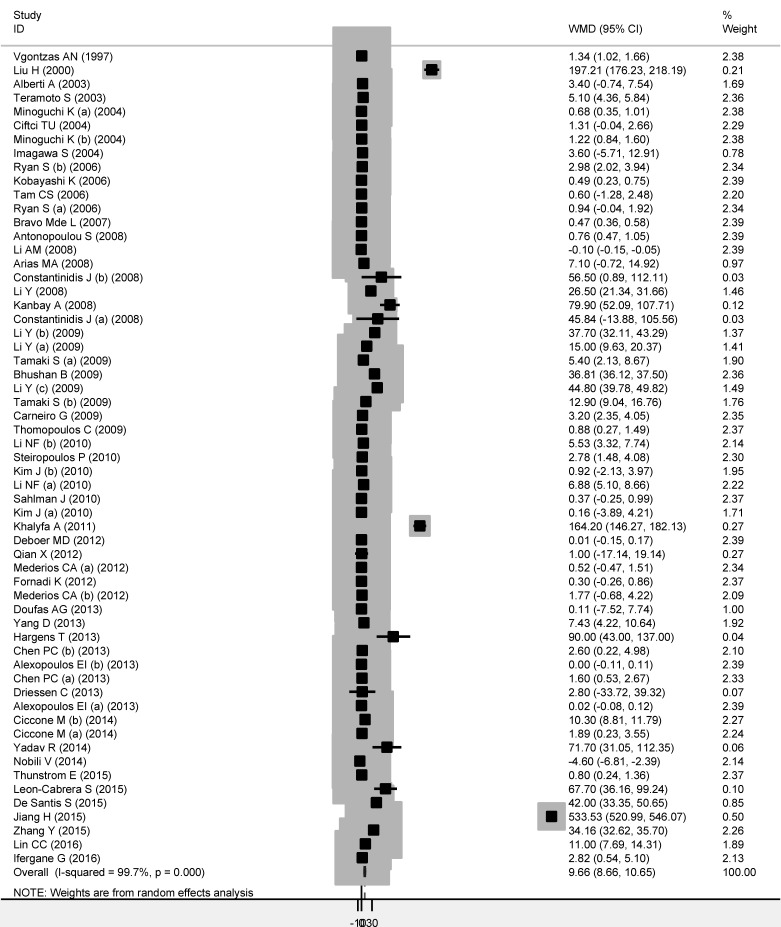
The forest plot for circulating TNF-alpha changes between OSAS patients and controls Abbreviations: WMD, weighted mean difference; 95% CI, 95% confidence interval; *I*-squared, inconsistency index. The x-axis represents the changes of circulating TNF-alpha between patients and controls in pg/mL.

**Figure 2:**
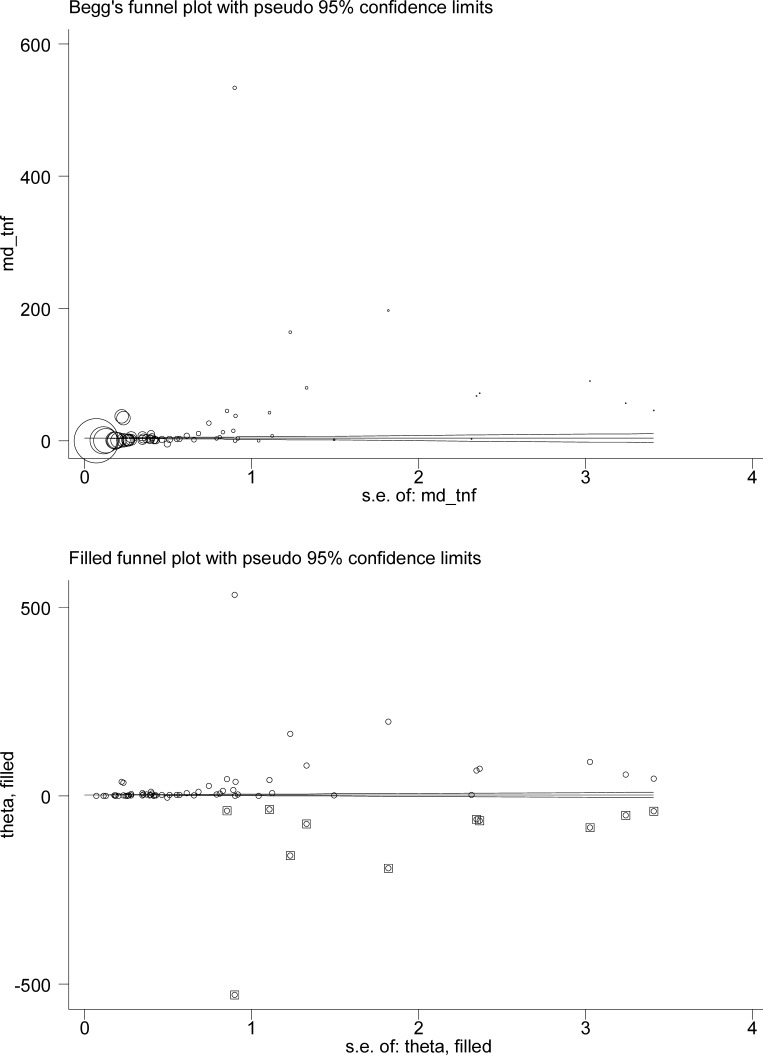
The Begg's (the upper) and filled (the lower) funnel plots for circulating TNF-alpha changes between OSAS patients and controls In the upper plot, the “md_tnf” in the y-axis is the mean difference of circulating TNF-alpha in pg/mL, and the “s.e. of: md_tnf” in the x-axis is the standard error of mean difference in circulating TNF-alpha. In the lower plot, the “theta” is the mean difference of circulating TNF-alpha in pg/mL, and the “s.e. of: theta” is the standard error of mean difference in circulating TNF-alpha.

When 59 qualified studies were pooled together, OSAS patients were observed to have a significantly higher level of circulating TNF-alpha than controls (WMD: 9.66 pg/mL, 95% CI: 8.66 to 11.24, *P* < 0.001) (Figure 1). Attention must be paid to this significant overall estimate, as heterogeneity across studies reached as high as 99.7% and the probability of Egger's test was 0.012. The filled funnel plot indicated that there were 11 missing studies with negative findings (Figure 2), and after adjusting for these missing studies, overall estimate was weakened but still significant (filled WMD: 2.63 pg/mL, 95% CI: 2.56 to 2.70, *P* < 0.001).

Stratified analyses according to age, gender, country, hypertension, diabetes mellitus, research type, matched condition, diagnostic criteria of controls, diagnostic criteria of OSAS and OSAS grade are shown in Table 2. In the analysis of studies involving underage individuals, there was no significant difference in circulating TNF-alpha between OSAS patients and controls (WMD: 0.00 pg/mL, 95% CI: -0.81 to 0.80, *P* = 0.991). After restricting analysis to males only, circulating TNF-alpha was significantly higher in OSAS patients than in controls (WMD: 1.52 pg/mL, 95% CI: 0.87 to 2.18, *P* < 0.001). This change was markedly reinforced in individuals free of hypertension and diabetes mellitus (WMD: 17.46 pg/mL, 95% CI: 15.70 to 19.21, *P* < 0.001), in studies with age-matched patients and controls (WMD: 28.57 pg/mL, 95% CI: 24.01 to 33.12, *P* < 0.001) and in studies adopting polysomnography to diagnose OSAS (WMD: 10.35 pg/mL, 95% CI: 9.29 to 11.41, *P* < 0.001).

**Table 2:** Stratified analyses on circulating TNF-alpha changes between OSAS patients and controls

Subgroups		No. of studies	WMD	95% CI	*P*	*I*^2^
Gender	Male	13	1.52	0.87 to 2.18	<0.001	87.9%
Age	Underage	7	0.00	−0.813 to 0.804	0.991	99.8%
Complication	Without Hypertension-DM	35	17.46	15.70 to 19.21	<0.001	99.8%
Match	Matched by age	23	28.57	24.01 to 33.12	<0.001	99.9%
Diagnosis	Polysomnography	51	10.35	9.29 to 11.41	<0.001	99.8%
Country	Brazil	3	1.85	−0.17 to 3.87	0.073	87.7%
	China	15	58.59	46.45 to 70.73	<0.001	99.9%
	Greece	7	0.48	0.13 to 0.83	0.007	88.9%
	Italy	5	9.32	1.71 to 16.93	0.016	98.0%
	Japan	7	2.99	1.70 to 4.29	<0.001	96.7%
	USA	5	6.00	2.75 to 9.24	<0.001	99.0%
Development	Developed countries	27	2.37	1.69 to 3.05	<0.001	97.2%
	Developing countries	32	17.17	15.47 to 18.87	<0.001	99.9%
Continent	Asian	25	29.84	26.21 to 33.47	<0.001	99.9%
	European	21	1.28	0.84 to 1.71	<0.001	95.4%
	North American	5	6.00	2.75 to 9.24	<0.001	99.0%
	South American	3	1.85	−0.17 to 3.87	0.073	99.7%
	Cross-continent	3	6.50	−0.58 to 13.58	0.072	93.7%
OSAS severity	All	23	22.48	20.11 to 24.84	<0.001	99.7%
	Mild	6	0.99	0.25 to 1.73	0.009	91.2%
	Mild-to-oderate	3	1.48	−0.11 to 3.06	0.068	74.5%
	Moderate	5	7.79	3.01 to 12.57	0.001	97.6%
	Moderate-to-severe	14	10.08	6.92 to 13.25	<0.001	99.9%
	Severe	8	8.85	4.40 to 13.31	<0.001	98.1%
Research type	Nested design	8	5.10	2.25 to 7.95	<0.001	95.9%
	Cross-sectional design	51	10.41	9.34 to 11.49	<0.001	99.7%

Abbreviations: WMD, weighted mean difference; 95% CI, 95% confidence interval; *I*^2^, inconsistency index.

In the following stratified analyses, only subgroups involving 3 or more studies were displayed. By country, OSAS patients vis-à-vis controls had remarkably high circulating TNF-alpha in China (WMD: 58.59 pg/mL, *P* < 0.001). When grouping studies by development, the changes in circulating TNF-alpha were strongly potentiated in developing countries (WMD: 17.17 pg/mL) than in developed countries (WMD: 2.37 pg/mL). Further by continent, the change was the highest in Asia (WMD: 29.84 pg/mL), followed by North America (WMD: 6.00 pg/mL) and Europe (WMD: 1.28 pg/mL). By research type, this change in cross-sectional case-control studies (WMD: 10.41 pg/mL) was overwhelming relative to nested case-control studies (WMD: 5.10 pg/mL). When studies were stratified by OSAS severity, the changes in circulating TNF-alpha between patients and controls increased gradually with the more severe grades of OSAS. In patients with mild, mild-to-moderate, moderate, moderate-to-severe and severe OSAS, circulating TNF-alpha was higher than respective controls by 0.99, 1.48. 7.79, 10.08 and 8.85 pg/mL. In spite of the above stratified analyses, there was no immediate improvement in between-study heterogeneity.

A meta-regression analysis was hence conducted to see the impact of other confounding factors on the changes of circulating TNF-alpha between OSAS patients and controls. After regressing all possible confounders as mentioned in the Methods, only abdomen circumference and IL-6 were found to exert a significant impact on the changes of circulating TNF-alpha (abdomen circumference: *P* < 0.001 in patients and *P* = 0.026 in controls; IL-6: *P* = 0.001 in patients and *P* = 0.003 in controls). No significance was found for the other confounders (data not shown). In view of this significant finding, correlation analysis was conducted to test the relationship of circulating TNF-alpha with abdomen circumference and IL-6. The correlation of circulating TNF-alpha with abdomen circumference was marginal (*P* = 0.078), while the correlation with IL-6 was remarkably significant (*P* < 0.001).

## DISCUSSION

On the basis of 59 studies and 4972 individuals, this meta-analysis aimed to quantify the changes of circulating TNF-alpha between OSAS patients and controls. Our results illustrated that circulating TNF-alpha was significantly higher in OSAS patients than in controls, and this difference became more pronounced with the more severe grades of OSAS, indicating that TNF-alpha might be a promising circulating biomarker for the development of OSAS.

There is strong evidence that TNF-alpha is a central regulator of inflammation, and its antagonists have proven to be efficacious in treating inflammatory diseases [59, 60]. OSAS is a chronic inflammatory disorder, and its presence can lead to the increased production of some inflammatory mediators in circulation, including TNF-alpha. An animal study found that the excessive sleepiness incurred by recurrent arousals during sleep might be due to the activation of TNF-alpha-depended inflammatory pathways [61, 62]. In addition, expression data showed that TNF-alpha was highly expressed in the heaviest OSAS patients relative to the less obese OSAS patients and non-apneic snorers [63]. The association of circulating TNF-alpha with OSAS risk has been widely evaluated, while no consensus exists in up-to-date literature [19, 51–54]. Based on these observations, it is reasonable to postulate that circulating TNF-alpha might be a clinical useful indicator for predicting OSAS risk. To shed some light on this postulation, we comprehensively analyzed the results of 59 studies through a meta-analysis and aimed to derive a reliable estimate between circulating TNF-alpha and OSAS.

A previous meta-analysis of 19 studies by Nadeem et al demonstrated that OSAS patients had higher circulating TNF-alpha than controls by 1.03 pg/mL, and this difference was confused by obvious heterogeneity that remained unexplored in their study [64]. The present meta-analysis by pooling the results of 59 studies confirmed and strengthened this significant difference by deriving an unbiased estimate of 2.63 pg/mL for circulating TNF-alpha in the trim-and-fill analysis. As with a majority of meta-analyses, we should be circumspect about the impact of between-study heterogeneity, as not every study's methodological and clinical aspects are identical [65]. In light of the differences in OSAS severity, research type, matched condition and so forth in the present meta-analysis, we can at least expound on some degree of heterogeneity, which accounted for part of conflicting findings in the literature. As it turns out, our stratified analyses demonstrated that the country, research type and OSAS severity might be possible sources of heterogeneity. It is worth mentioning that with the more severe grades of OSAS defined by AHI, circulating TNF-alpha was much higher in patients than in controls. Although the observational nature of all involved studies in this meta-analysis precluded the causal-effect exploration between circulating TNF-alpha and OSAS, our findings may provide indirect evidence that TNF-alpha might be a promising circulating biomarker for the development of OSAS. We concede that whether elevated circulating TNF-alpha is the cause or the effect of OSAS remains an open question. In the future, clinical trials are warranted to dissect this relation.

In spite of clear strengths including a large number of qualified studies and a comprehensive exploration on heterogeneity, it should be realized that there are several limitations to association studies included in this meta-analysis. First, selection bias might be possible given that only English articles were indexed. Although there was a significant probability of publication bias, the filled effect estimate after adjusting for missing studies was still significant in circulating TNF-alpha between OSAS patients and controls. Second, the results of this meta-analysis were based on 59 studies, while the total sample was not large enough. The power to reject the null hypothesis is very limited in some subgroup analyses. Third, between-study heterogeneity cannot be fully accounted for, in spite of a wide panel of stratified analyses conducted. It will be encouraging to explore the other sources of methodological and clinical aspects to mitigate heterogeneity. Moreover, this meta-analysis was undertaken with summary data, and to thoroughly account for heterogeneity one usually needs to perform a meta-analysis based on individual participant data, which are not always feasible. Fourth, the impact of obesity on the relationship between circulating TNF-alpha and OSAS cannot be solved due to the lack of necessary data, although it is increasingly recognized that obesity is an established risk factor for OSAS.

In sum, this meta-analysis of 59 studies and 4972 individuals demonstrated that circulating TNF-alpha was significantly higher in OSAS patients than in controls, and this difference became more pronounced with the more severe grades of OSAS, indicating that TNF-alpha might be a promising circulating biomarker for the development of OSAS. Our results, as are of consequence, deserve to be tested through relevant biological means and validated in large, well-designed prospective studies.

## MATERIALS AND METHODS

This is a systematic meta-analysis on observational data, and its conduct complies with the guidelines enacted by the Meta-analysis Of Observational Studies in Epidemiology (MOOSE) group [66].

Using public databases of PubMed, Embase and Web of Science, articles that reported the changes of circulating TNF-alpha between OSAS patients and controls were indexed on November 3, 2016. Research content was confined to materials written in English language only. Included articles had to meet the following criteria: (i) OSAS as the clinical endpoint diagnosed by standard methods; (ii) case-control study design; (iii) availability of serum or plasma TNF-alpha levels expressed as mean or median value along with standard deviation or standard error or 95% confidence interval (95% CI) or interquartile range or range in both OSAS patients and controls.

Exclusion process of candidate articles was accomplished with two steps: first, the title and abstract were reviewed to remove articles that were clearly irrelevant, such as animal experiments or clinical interventions; second, the full text of the remaining articles was evaluated according to the inclusive criteria, and meanwhile the reference list of each qualified article was also inspected to avoid possible loss of candidates. Two reviewers (Qingsheng Li and Xin Zheng) independently implemented literature search and exclusion process, and they settled all inconsistencies by discussion.

The following data were drawn from each qualified article: the first author's surname, publication year, country where study samples were collected from, research type, diagnostic criteria and method of OSAS, sample size, matched condition, age, gender, body mass index (BMI), abdomen circumference, neck circumference, smoking, hypertension, diabetes mellitus, systolic blood pressure (SBP), diastolic blood pressure (DBP), total cholesterol, triglycerides, high-density lipoprotein cholesterol (HDLC), low-density lipoprotein cholesterol (LDLC), glucose, C-reaction protein (CRP), interleukin-6 (IL-6), rapid eye movement (REM), sleep efficiency, apnea-hypopnea index (AHI), oxygen desaturation index (ODI), arterial hemoglobin saturation (SaO_2_), SaO_2_ < 90%, Epworth sleepiness scale (ESS) and serum or plasma TNF-alpha. Information-drawing process was independently implemented by two reviewers (Qingsheng Li and Xin Zheng), who resolved any disagreement by consensus.

Statistical analyses were handled using the STATA software (11^th^ version). The changes of circulating TNF-alpha were expressed with the weighted mean difference (WMD) along with its 95% CI. Heterogeneity is measured by the *I*^2^ statistic, which is calculated as 100%×(Q -d.f.)/Q (here Q is the Cochran's heterogeneity statistic and d.f. is the degree of freedom) and describes the percentage of total variation across studies that results from heterogeneity rather than from chance [67]. In case of no heterogeneity (the *I*^2^ statistic < 50%), a fixed-effects model was adopted to calculate the WMD and 95% CI. Otherwise, a random-effects model was adopted.

Possible causes of heterogeneity were looked for by stratified analyses and meta-regression analyses. Stratified factors included age, gender, country, hypertension, diabetes mellitus, research type, matched condition, diagnostic criteria of controls, diagnostic criteria of OSAS patients and OSAS grade. Other variables modeled in meta-regression analyses included age, gender, BMI, abdomen circumference, neck circumference, smoking, hypertension, diabetes mellitus, SBP, DBP, total cholesterol, triglycerides, HDLC, LDLC, glucose, CRP, IL-6, REM, sleep efficiency, AHI, ODI, SaO_2_, SaO_2_ < 90% and ESS.

The Begg's funnel plot was created to illustrate the likelihood of publication bias, which was statistically evaluated by the Egger's test. In addition, a filled funnel plot by the fill-and-trim method was also created to determine the number of missing studies with negative findings and filled effect estimates were derived accordingly.

## SUPPLEMENTARY MATERIALS TABLE


