# Supplementary material for: Tumor necrosis factor alpha is a promising circulating biomarker for the development of obstructive sleep apnea syndrome: a meta-analysis
Source: Oncotarget. 2017 Feb 8;8(16):27616–26. doi: 10.18632/oncotarget.15203 (PMC5432362; doi:10.18632/oncotarget.15203)
Supplement: Supplementary file 1 [file oncotarget-08-27616-s001.docx]

**Supporting Table 1. The other baseline characteristics of 59 studies in the present meta-analysis**

| **First author** | **Year** | **Male** | **Underage** | **W/O Hypert-DM** | **Matched by age** | **OSAS diagnosis** | **Diagnostic criteria** | | **Abdomen circumference (cm)** | | **Neck circumference (cm)** | |
| --- | --- | --- | --- | --- | --- | --- | --- | --- | --- | --- | --- | --- |
|  |  |  |  |  |  |  | **Patients** | **Controls** | **Patients** | **Controls** | **Patients** | **Controls** |
| Vgontzas AN | 1997 |  |  |  |  | polysomnography | AHI>5 | AHI<5 | N.R. | N.R. | N.R. | N.R. |
| Liu H | 2000 |  |  |  |  | polysomnography | AHI>5 | AHI<5 | N.R. | N.R. | N.R. | N.R. |
| Teramoto S | 2003 |  |  | YES | YES | polysomnography | AHI>5 | AHI<5 | N.R. | N.R. | N.R. | N.R. |
| Alberti A | 2003 |  |  |  | YES | polysomnography | AHI>10 | AHI<5 | N.R. | N.R. | N.R. | N.R. |
| Minoguchi K (a) | 2004 | YES |  |  |  | polysomnography | AHI>5 & AHI<20 | AHI<5 | N.R. | N.R. | N.R. | N.R. |
| Minoguchi K (b) | 2004 | YES |  |  |  | polysomnography | AHI>20 | AHI<5 | N.R. | N.R. | N.R. | N.R. |
| Imagawa S | 2004 |  |  | YES |  | N.R. | AHI>30 | AHI<5 | N.R. | N.R. | N.R. | N.R. |
| Ciftci TU | 2004 | YES |  | YES | YES | polysomnography | AHI>5 | AHI<5 | N.R. | N.R. | 41.79 | 39.52 |
| Tam CS | 2006 |  | YES | YES |  | respiratory disturbance index | RDI>1 | RDI<1 | N.R. | N.R. | N.R. | N.R. |
| Ryan S (a) | 2006 | YES |  | YES | YES | polysomnography | AHI>5 & AHI<30 | AHI<5 | N.R. | N.R. | N.R. | N.R. |
| Ryan S (b) | 2006 | YES |  | YES | YES | polysomnography | AHI>30 | AHI<5 | N.R. | N.R. | N.R. | N.R. |
| Kobayashi K | 2006 |  |  |  |  | polysomnography | AHI>30 | AHI<5 | N.R. | N.R. | N.R. | N.R. |
| Bravo Mde L | 2007 | YES |  | YES | YES | polysomnography | AHI>20 | AHI<5 | N.R. | N.R. | N.R. | N.R. |
| Li Y | 2008 |  |  | YES |  | polysomnography | AHI>5 | AHI<5 | 94.20 | 80.70 | 39.80 | 36.40 |
| Li AM | 2008 |  |  |  |  | polysomnography | AHI>1 | AHI<1 | N.R. | N.R. | N.R. | N.R. |
| Kanbay A | 2008 |  |  |  |  | polysomnography | AHI>5 | AHI<5 | N.R. | N.R. | N.R. | N.R. |
| Constantinidis J (a) | 2008 | YES |  |  |  | polysomnography | AHI>5 | AHI<5 | N.R. | N.R. | N.R. | N.R. |
| Constantinidis J (b) | 2008 | YES |  |  |  | polysomnography | AHI>5 | AHI<5 | N.R. | N.R. | N.R. | N.R. |
| Arias MA | 2008 | YES |  | YES | YES | respiratory recording device | AHI>10 | AHI<5 | N.R. | N.R. | N.R. | N.R. |
| Antonopoulou S | 2008 |  |  | YES | YES | polysomnography | AHI>10 | AHI<5 | N.R. | N.R. | N.R. | N.R. |
| Thomopoulos C | 2009 |  |  |  |  | polysomnography | AHI>5 | AHI<5 | 105.60 | 105.50 | N.R. | N.R. |
| Tamaki S (a) | 2009 |  |  | YES |  | polysomnography | AHI>10 & AHI<30 | AHI<10 | N.R. | N.R. | N.R. | N.R. |
| Tamaki S (b) | 2009 |  |  | YES |  | polysomnography | AHI>30 | AHI<10 | N.R. | N.R. | N.R. | N.R. |
| Li Y (a) | 2009 |  |  | YES |  | polysomnography | AHI>5 & AHI<20 | AHI<5 | N.R. | N.R. | N.R. | N.R. |
| Li Y (b) | 2009 |  |  | YES |  | polysomnography | AHI>20 & AHI<40 | AHI<5 | N.R. | N.R. | N.R. | N.R. |
| Li Y (c) | 2009 |  |  | YES |  | polysomnography | AHI>30 | AHI<5 | N.R. | N.R. | N.R. | N.R. |
| Carneiro G | 2009 | YES |  |  | YES | polysomnography | AHI>5 | AHI<5 | N.R. | N.R. | N.R. | N.R. |
| Bhushan B | 2009 |  |  | YES | YES | polysomnography | AHI>10 | AHI<5 | 105.00 | 102.80 | 39.60 | 37.90 |
| Steiropoulos P | 2010 |  |  | YES | YES | polysomnography | AHI>15 | AHI<15 | 118.40 | 114.70 | 44.10 | 40.50 |
| Sahlman J | 2010 |  |  |  |  | polysomnography | AHI>5 & AHI<15 | AHI<5 | N.R. | N.R. | N.R. | N.R. |
| Li NF (a) | 2010 |  |  | YES | YES | polysomnography | AHI>10 | AHI<10 | 99.04 | 94.58 | 39.86 | 38.20 |
| Li NF (b) | 2010 |  |  |  | YES | polysomnography | AHI>10 | AHI<10 | 102.19 | 98.24 | 40.58 | 39.74 |
| Kim J (a) | 2010 |  |  | YES |  | polysomnography | AHI>5 & AHI<20 | AHI<5 | N.R. | N.R. | N.R. | N.R. |
| Kim J (b) | 2010 |  |  | YES |  | polysomnography | AHI>20 | AHI<5 | N.R. | N.R. | N.R. | N.R. |
| Khalyfa A | 2011 |  | YES | YES | YES | polysomnography | AHI>1 | AHI<1 | N.R. | N.R. | N.R. | N.R. |
| Qian X | 2012 | YES |  |  | YES | polysomnography | AHI>30 | AHI<5 | N.R. | N.R. | N.R. | N.R. |
| Mederios CA (a) | 2012 |  |  |  | YES | polysomnography | AHI>5 & AHI<30 | AHI<5 | N.R. | N.R. | 40.83 | 39.53 |
| Mederios CA (b) | 2012 |  |  |  | YES | polysomnography | AHI>30 | AHI<5 | N.R. | N.R. | 40.76 | 39.53 |
| Deboer MD | 2012 |  | YES | YES |  | polysomnography | AHI>1 | AHI<1 | N.R. | N.R. | N.R. | N.R. |
| Fornadi K | 2012 |  |  |  |  | polysomnography | AHI>5 | AHI<5 | N.R. | N.R. | N.R. | N.R. |
| Yang D | 2013 |  |  |  |  | polysomnography | AHI>5 | AHI<5 | 97.72 | 94.98 | 41.00 | 39.78 |
| Hargens T | 2013 | YES |  | YES |  | Embletta | AHI>5 | AHI<5 | N.R. | N.R. | 40.70 | 36.30 |
| Driessen C | 2013 |  | YES |  |  | Embletta | AHI>1 | AHI<1 | N.R. | N.R. | N.R. | N.R. |
| Doufas AG | 2013 | YES |  | YES |  | polysomnography | AHI>5 | AHI<5 | N.R. | N.R. | N.R. | N.R. |
| Chen PC (a) | 2013 |  |  | YES | YES | polysomnography | AHI>5 & AHI<15 | AHI<5 | 90.70 | 84.10 | 36.60 | 34.80 |
| Chen PC (b) | 2013 |  |  | YES | YES | polysomnography | AHI>15 & AHI<30 | AHI<5 | 89.70 | 84.10 | 39.00 | 34.80 |
| Alexopoulos EI (a) | 2013 |  | YES | YES |  | polysomnography | AHI>1 & AHI<5 | AHI<1 | N.R. | N.R. | N.R. | N.R. |
| Alexopoulos EI (b) | 2013 |  | YES | YES |  | polysomnography | AHI>5 | AHI<1 | N.R. | N.R. | N.R. | N.R. |
| Yadav R | 2014 |  |  |  | YES | polysomnography | AHI>15 | AHI<5 | 143.00 | 140.00 | N.R. | N.R. |
| Nobili V | 2014 |  | YES |  |  | polysomnography | AHI>1 | AHI<1 | 90.00 | 88.70 | N.R. | N.R. |
| Ciccone M (a) | 2014 |  |  | YES | YES | PM | AHI>5 & AHI<15 | AHI<5 | N.R. | N.R. | 40.03 | 40.12 |
| Ciccone M (b) | 2014 |  |  | YES | YES | PM | AHI>15 | AHI<5 | N.R. | N.R. | 40.61 | 40.12 |
| Zhang Y | 2015 |  |  | YES |  | polysomnography | AHI>15 | AHI<5 | 102.39 | 88.56 | 41.54 | 38.20 |
| Thunstrom E | 2015 |  |  |  |  | cardiorespiratory polygraphy | AHI>15 | AHI<5 | 98.10 | 91.50 | N.R. | N.R. |
| Leon-Cabrera S | 2015 |  |  | YES |  | polysomnography | AHI>15 | AHI<5 | 124.20 | 84.30 | N.R. | N.R. |
| Jiang H | 2015 |  |  | YES | YES | polysomnography | AHI>5 | AHI<5 | N.R. | N.R. | 40.70 | 36.80 |
| De Santis S | 2015 |  |  | YES |  | polysomnography | AHI>5 | AHI<5 | N.R. | N.R. | N.R. | N.R. |
| Lin CC | 2016 |  |  | YES |  | polysomnography | AHI>5 | AHI<5 | N.R. | N.R. | N.R. | N.R. |
| Ifergane G | 2016 |  |  |  |  | polysomnography | AHI>15 | AHI<15 | N.R. | N.R. | N.R. | N.R. |

**(Cont’d)**

| **Smoking** | | **SBP (mmHg)** | | **DBP (mmHg)** | | **TC (mg/dL)** | | **Triglycerides (mg/dL)** | | **HDLC (mg/dL)** | | **LDLC (mg/dL)** | | **Glucose (mmol/L)** | | **CRP (mg/dL)** | |
| --- | --- | --- | --- | --- | --- | --- | --- | --- | --- | --- | --- | --- | --- | --- | --- | --- | --- |
| **Patients** | **Controls** | **Patients** | **Controls** | **Patients** | **Controls** | **Patients** | **Controls** | **Patients** | **Controls** | **Patients** | **Controls** | **Patients** | **Controls** | **Patients** | **Controls** | **Patients** | **Controls** |
| N.R. | N.R. | N.R. | N.R. | N.R. | N.R. | N.R. | N.R. | N.R. | N.R. | N.R. | N.R. | N.R. | N.R. | N.R. | N.R. | N.R. | N.R. |
| N.R. | N.R. | N.R. | N.R. | N.R. | N.R. | N.R. | N.R. | N.R. | N.R. | N.R. | N.R. | N.R. | N.R. | N.R. | N.R. | N.R. | N.R. |
| N.R. | N.R. | N.R. | N.R. | N.R. | N.R. | N.R. | N.R. | N.R. | N.R. | N.R. | N.R. | N.R. | N.R. | N.R. | N.R. | 0.3 | 0.1 |
| 0.53 | 0.00 | N.R. | N.R. | N.R. | N.R. | N.R. | N.R. | N.R. | N.R. | N.R. | N.R. | N.R. | N.R. | N.R. | N.R. | N.R. | N.R. |
| 0.00 | 0.00 | N.R. | N.R. | N.R. | N.R. | 197.1 | 192.9 | 132.8 | 136.1 | 36.1 | 40.1 | 130.1 | 124.7 | N.R. | N.R. | N.R. | N.R. |
| 0.00 | 0.00 | N.R. | N.R. | N.R. | N.R. | 205.8 | 192.9 | 175.2 | 136.1 | 39.5 | 40.1 | 175.2 | 124.7 | N.R. | N.R. | N.R. | N.R. |
| N.R. | N.R. | N.R. | N.R. | N.R. | N.R. | N.R. | N.R. | N.R. | N.R. | N.R. | N.R. | N.R. | N.R. | N.R. | N.R. | N.R. | N.R. |
| N.R. | N.R. | N.R. | N.R. | N.R. | N.R. | N.R. | N.R. | N.R. | N.R. | N.R. | N.R. | N.R. | N.R. | N.R. | N.R. | N.R. | N.R. |
| 0.00 | 0.00 | N.R. | N.R. | N.R. | N.R. | N.R. | N.R. | N.R. | N.R. | N.R. | N.R. | N.R. | N.R. | N.R. | N.R. | 0.8 | 0.8 |
| 0.37 | 0.30 | 128.0 | 131.0 | 81.0 | 82.0 | 201.1 | 204.9 | N.R. | N.R. | 39.8 | 39.4 | 127.2 | 138.1 | N.R. | N.R. | N.R. | N.R. |
| 0.39 | 0.30 | 135.0 | 131.0 | 86.0 | 82.0 | 204.9 | 204.9 | N.R. | N.R. | 38.7 | 39.4 | 134.2 | 138.1 | N.R. | N.R. | N.R. | N.R. |
| 0.46 | 0.38 | N.R. | N.R. | N.R. | N.R. | N.R. | N.R. | N.R. | N.R. | N.R. | N.R. | N.R. | N.R. | N.R. | N.R. | N.R. | N.R. |
| 0.41 | 0.00 | 146.2 | 127.0 | 93.3 | 78.3 | 222.2 | 220.7 | 153.1 | 132.9 | 56.3 | 51.0 | 139.8 | 143.1 | 5.8 | 5.5 | N.R. | N.R. |
| 0.00 | 0.00 | N.R. | N.R. | N.R. | N.R. | N.R. | N.R. | N.R. | N.R. | N.R. | N.R. | N.R. | N.R. | N.R. | N.R. | N.R. | N.R. |
| N.R. | N.R. | 122.0 | 114.0 | 68.0 | 66.0 | N.R. | N.R. | N.R. | N.R. | N.R. | N.R. | N.R. | N.R. | N.R. | N.R. | N.R. | N.R. |
| N.R. | N.R. | 135.4 | 131.5 | 80.2 | 78.3 | N.R. | N.R. | N.R. | N.R. | N.R. | N.R. | N.R. | N.R. | N.R. | N.R. | N.R. | N.R. |
| N.R. | N.R. | N.R. | N.R. | N.R. | N.R. | N.R. | N.R. | N.R. | N.R. | N.R. | N.R. | N.R. | N.R. | N.R. | N.R. | N.R. | N.R. |
| N.R. | N.R. | N.R. | N.R. | N.R. | N.R. | N.R. | N.R. | N.R. | N.R. | N.R. | N.R. | N.R. | N.R. | N.R. | N.R. | N.R. | N.R. |
| 0.37 | 0.27 | 126.0 | 122.0 | 79.0 | 78.0 | N.R. | N.R. | N.R. | N.R. | N.R. | N.R. | N.R. | N.R. | N.R. | N.R. | N.R. | N.R. |
| N.R. | N.R. | N.R. | N.R. | N.R. | N.R. | N.R. | N.R. | N.R. | N.R. | N.R. | N.R. | N.R. | N.R. | N.R. | N.R. | N.R. | N.R. |
| 0.50 | 0.53 | 150.7 | 148.0 | 97.2 | 95.9 | 170.1 | 170.1 | 150.6 | 141.7 | 46.4 | 46.4 | 146.9 | 150.8 | N.R. | N.R. | 3.6 | 2.5 |
| N.R. | N.R. | N.R. | N.R. | N.R. | N.R. | N.R. | N.R. | N.R. | N.R. | N.R. | N.R. | N.R. | N.R. | N.R. | N.R. | N.R. | N.R. |
| N.R. | N.R. | N.R. | N.R. | N.R. | N.R. | N.R. | N.R. | N.R. | N.R. | N.R. | N.R. | N.R. | N.R. | N.R. | N.R. | N.R. | N.R. |
| 0.00 | 0.00 | N.R. | N.R. | N.R. | N.R. | N.R. | N.R. | N.R. | N.R. | N.R. | N.R. | N.R. | N.R. | N.R. | N.R. | N.R. | N.R. |
| 0.00 | 0.00 | N.R. | N.R. | N.R. | N.R. | N.R. | N.R. | N.R. | N.R. | N.R. | N.R. | N.R. | N.R. | N.R. | N.R. | N.R. | N.R. |
| 0.00 | 0.00 | N.R. | N.R. | N.R. | N.R. | N.R. | N.R. | N.R. | N.R. | N.R. | N.R. | N.R. | N.R. | N.R. | N.R. | N.R. | N.R. |
| 0.00 | 0.00 | N.R. | N.R. | N.R. | N.R. | N.R. | N.R. | N.R. | N.R. | N.R. | N.R. | N.R. | N.R. | N.R. | N.R. | 0.8 | 0.9 |
| 0.00 | 0.00 | 135.3 | 132.6 | 89.6 | 85.7 | 194.2 | 188.9 | 170.0 | 160.1 | 44.6 | 44.4 | N.R. | N.R. | 6.3 | 5.8 | N.R. | N.R. |
| 0.00 | 0.00 | 131.1 | 131.2 | 80.6 | 80.2 | N.R. | N.R. | N.R. | N.R. | N.R. | N.R. | N.R. | N.R. | N.R. | N.R. | 0.6 | 0.5 |
| 0.24 | 0.25 | N.R. | N.R. | N.R. | N.R. | 185.6 | 193.3 | 150.6 | 132.9 | 42.5 | 46.4 | N.R. | N.R. | 6.2 | 5.9 | 1.7 | 1.3 |
| N.R. | N.R. | N.R. | N.R. | N.R. | N.R. | N.R. | N.R. | N.R. | N.R. | N.R. | N.R. | N.R. | N.R. | N.R. | N.R. | N.R. | N.R. |
| N.R. | N.R. | N.R. | N.R. | N.R. | N.R. | N.R. | N.R. | N.R. | N.R. | N.R. | N.R. | N.R. | N.R. | N.R. | N.R. | N.R. | N.R. |
| N.R. | N.R. | N.R. | N.R. | N.R. | N.R. | N.R. | N.R. | N.R. | N.R. | N.R. | N.R. | N.R. | N.R. | N.R. | N.R. | N.R. | N.R. |
| N.R. | N.R. | N.R. | N.R. | N.R. | N.R. | N.R. | N.R. | N.R. | N.R. | N.R. | N.R. | N.R. | N.R. | N.R. | N.R. | N.R. | N.R. |
| N.R. | N.R. | N.R. | N.R. | N.R. | N.R. | N.R. | N.R. | N.R. | N.R. | N.R. | N.R. | N.R. | N.R. | N.R. | N.R. | N.R. | N.R. |
| N.R. | N.R. | N.R. | N.R. | N.R. | N.R. | N.R. | N.R. | N.R. | N.R. | 36.7 | 37.5 | 104.8 | 104.0 | N.R. | N.R. | 0.3 | 0.2 |
| N.R. | N.R. | N.R. | N.R. | N.R. | N.R. | N.R. | N.R. | N.R. | N.R. | N.R. | N.R. | N.R. | N.R. | 6.7 | N.R. | N.R. | N.R. |
| N.R. | N.R. | N.R. | N.R. | N.R. | N.R. | N.R. | N.R. | N.R. | N.R. | N.R. | N.R. | N.R. | N.R. | 8.0 | N.R. | N.R. | N.R. |
| N.R. | N.R. | N.R. | N.R. | N.R. | N.R. | N.R. | N.R. | N.R. | N.R. | N.R. | N.R. | N.R. | N.R. | 5.7 | 5.3 | 1.7 | 2.0 |
| N.R. | N.R. | N.R. | N.R. | N.R. | N.R. | N.R. | N.R. | N.R. | N.R. | N.R. | N.R. | N.R. | N.R. | N.R. | N.R. | 3.8 | 2.8 |
| 0.48 | 0.48 | N.R. | N.R. | N.R. | N.R. | N.R. | N.R. | N.R. | N.R. | N.R. | N.R. | N.R. | N.R. | N.R. | N.R. | N.R. | N.R. |
| 0.00 | 0.00 | N.R. | N.R. | N.R. | N.R. | N.R. | N.R. | N.R. | N.R. | N.R. | N.R. | N.R. | N.R. | 4.8 | 4.7 | N.R. | N.R. |
| N.R. | N.R. | N.R. | N.R. | N.R. | N.R. | N.R. | N.R. | N.R. | N.R. | N.R. | N.R. | N.R. | N.R. | N.R. | N.R. | 0.5 | 0.4 |
| N.R. | N.R. | N.R. | N.R. | N.R. | N.R. | N.R. | N.R. | N.R. | N.R. | N.R. | N.R. | N.R. | N.R. | 5.1 | 5.1 | N.R. | N.R. |
| 0.00 | 0.00 | 127.0 | 117.0 | 75.0 | 71.0 | 197.0 | 170.0 | 157.0 | 137.0 | N.R. | N.R. | N.R. | N.R. | 5.3 | 4.9 | 0.3 | 0.1 |
| 0.00 | 0.00 | 127.0 | 117.0 | 77.0 | 71.0 | 215.0 | 170.0 | 156.0 | 137.0 | N.R. | N.R. | N.R. | N.R. | 5.5 | 4.9 | 0.7 | 0.1 |
| 0.00 | 0.00 | N.R. | N.R. | N.R. | N.R. | N.R. | N.R. | N.R. | N.R. | N.R. | N.R. | N.R. | N.R. | N.R. | N.R. | N.R. | N.R. |
| 0.00 | 0.00 | N.R. | N.R. | N.R. | N.R. | N.R. | N.R. | N.R. | N.R. | N.R. | N.R. | N.R. | N.R. | N.R. | N.R. | N.R. | N.R. |
| 0.30 | 0.20 | 139.0 | 139.0 | 76.0 | 75.0 | 185.6 | 204.9 | 150.6 | 141.7 | 50.3 | 50.3 | 123.7 | 123.7 | N.R. | N.R. | 6.9 | 7.9 |
| 0.00 | 0.00 | 114.0 | 113.0 | 68.0 | 68.0 | 165.0 | 157.0 | 118.0 | 103.0 | 41.0 | 44.0 | N.R. | N.R. | 4.9 | 4.9 | 1.5 | 1.6 |
| 0.00 | 0.00 | 124.6 | 124.4 | 77.3 | 78.6 | N.R. | N.R. | N.R. | N.R. | N.R. | N.R. | N.R. | N.R. | N.R. | N.R. | 1.3 | 1.1 |
| 0.00 | 0.00 | 127.6 | 124.4 | 80.2 | 78.6 | N.R. | N.R. | N.R. | N.R. | N.R. | N.R. | N.R. | N.R. | N.R. | N.R. | 1.8 | 1.1 |
| N.R. | N.R. | 140.4 | 120.9 | 95.1 | 73.8 | 179.0 | 132.6 | 217.0 | 194.9 | N.R. | N.R. | 100.2 | 101.7 | 5.5 | 5.1 | N.R. | N.R. |
| 0.15 | 0.22 | N.R. | N.R. | N.R. | N.R. | N.R. | N.R. | N.R. | N.R. | N.R. | N.R. | N.R. | N.R. | N.R. | N.R. | 1.9 | 0.9 |
| N.R. | N.R. | N.R. | N.R. | N.R. | N.R. | 201.6 | 192.3 | 227.3 | 121.8 | N.R. | N.R. | N.R. | N.R. | 6.7 | 5.0 | N.R. | N.R. |
| N.R. | N.R. | N.R. | N.R. | N.R. | N.R. | N.R. | N.R. | N.R. | N.R. | N.R. | N.R. | N.R. | N.R. | N.R. | N.R. | N.R. | N.R. |
| N.R. | N.R. | N.R. | N.R. | N.R. | N.R. | N.R. | N.R. | N.R. | N.R. | N.R. | N.R. | N.R. | N.R. | N.R. | N.R. | N.R. | N.R. |
| 0.00 | 0.00 | 122.9 | 120.8 | 73.8 | 71.8 | 203.9 | 202.6 | 213.3 | 207.7 | N.R. | N.R. | N.R. | N.R. | N.R. | N.R. | N.R. | N.R. |
| N.R. | N.R. | 152.6 | 159.5 | 84.4 | 88.2 | N.R. | N.R. | N.R. | N.R. | N.R. | N.R. | 99.9 | 118.8 | 7.1 | 7.2 | N.R. | N.R. |

**(Cont’d)**

| **IL-6 (pg/dL)** | | **REM (%)** | | **Sleep efficiency (%)** | | **ODI (events/h)** | | **SaO2 (%)** | | **SaO2<90% (%)** | | **ESS** | |
| --- | --- | --- | --- | --- | --- | --- | --- | --- | --- | --- | --- | --- | --- |
| **Patients** | **Controls** | **Patients** | **Controls** | **Patients** | **Controls** | **Patients** | **Controls** | **Patients** | **Controls** | **Patients** | **Controls** | **Patients** | **Controls** |
| 3.3 | 1.0 | 12.7 | 24.1 | N.R. | N.R. | N.R. | N.R. | 70.1 | 96.7 | N.R. | N.R. | N.R. | N.R. |
| 50.7 | 12.7 | N.R. | N.R. | N.R. | N.R. | N.R. | N.R. | 89.1 | 94.4 | 15.5 | 2.1 | N.R. | N.R. |
| 7.6 | 3.5 | N.R. | N.R. | N.R. | N.R. | N.R. | N.R. | N.R. | N.R. | N.R. | N.R. | N.R. | N.R. |
| 6.4 | 4.9 | N.R. | N.R. | N.R. | N.R. | N.R. | N.R. | 83.3 | N.R. | N.R. | N.R. | 11.7 | N.R. |
| N.R. | N.R. | N.R. | N.R. | N.R. | N.R. | N.R. | N.R. | 82.8 | 97.9 | 4.0 | 0.0 | 10.2 | 4.5 |
| N.R. | N.R. | N.R. | N.R. | N.R. | N.R. | N.R. | N.R. | 68.4 | 97.9 | 42.2 | 0.0 | 13.5 | 4.5 |
| 38.5 | 24.4 | N.R. | N.R. | N.R. | N.R. | N.R. | N.R. | N.R. | N.R. | N.R. | N.R. | N.R. | N.R. |
| 11.7 | 6.1 | N.R. | N.R. | N.R. | N.R. | N.R. | N.R. | N.R. | N.R. | N.R. | N.R. | 12.1 | 11.4 |
| 5.3 | 4.8 | N.R. | N.R. | 79.8 | N.R. | N.R. | N.R. | N.R. | N.R. | N.R. | N.R. | N.R. | N.R. |
| N.R. | N.R. | N.R. | N.R. | N.R. | N.R. | 15.0 | 2.0 | 93.4 | 94.0 | N.R. | N.R. | 11.0 | 8.0 |
| N.R. | N.R. | N.R. | N.R. | N.R. | N.R. | 56.6 | 2.0 | 92.4 | 94.0 | N.R. | N.R. | 15.0 | 8.0 |
| N.R. | N.R. | N.R. | N.R. | N.R. | N.R. | N.R. | N.R. | N.R. | N.R. | N.R. | N.R. | N.R. | N.R. |
| 2.4 | 1.1 | N.R. | N.R. | N.R. | N.R. | N.R. | N.R. | 89.4 | 93.5 | N.R. | N.R. | 5.3 | 2.1 |
| 65.3 | 37.5 | N.R. | N.R. | N.R. | N.R. | N.R. | N.R. | 90.1 | 93.0 | N.R. | N.R. | N.R. | N.R. |
| 0.1 | 0.1 | N.R. | N.R. | N.R. | N.R. | N.R. | N.R. | 74.0 | 88.0 | N.R. | N.R. | N.R. | N.R. |
| N.R. | N.R. | N.R. | N.R. | 73.95 | 75.12 | N.R. | N.R. | 75.9 | 86.6 | N.R. | N.R. | N.R. | N.R. |
| 0.8 | 0.4 | N.R. | N.R. | N.R. | N.R. | N.R. | N.R. | N.R. | N.R. | N.R. | N.R. | N.R. | N.R. |
| 0.5 | 0.1 | N.R. | N.R. | N.R. | N.R. | N.R. | N.R. | N.R. | N.R. | N.R. | N.R. | N.R. | N.R. |
| 4.2 | 3.6 | N.R. | N.R. | N.R. | N.R. | 44.6 | 4.7 | 91.0 | 94.0 | 18.4 | 0.0 | N.R. | N.R. |
| N.R. | N.R. | N.R. | N.R. | N.R. | N.R. | 41.0 | 1.4 | 88.0 | 95.0 | 48.0 | 1.7 | 8.0 | 0.6 |
| 1.4 | 0.4 | N.R. | N.R. | N.R. | N.R. | N.R. | N.R. | 80.2 | 92.7 | N.R. | N.R. | N.R. | N.R. |
| N.R. | N.R. | N.R. | N.R. | N.R. | N.R. | 14.5 | 5.5 | N.R. | N.R. | 10.9 | 0.1 | N.R. | N.R. |
| N.R. | N.R. | N.R. | N.R. | N.R. | N.R. | 51.7 | 5.5 | N.R. | N.R. | 26.6 | 0.1 | N.R. | N.R. |
| 55.2 | 37.5 | N.R. | N.R. | N.R. | N.R. | N.R. | N.R. | 91.0 | 93.0 | N.R. | N.R. | N.R. | N.R. |
| 73.5 | 37.5 | N.R. | N.R. | N.R. | N.R. | N.R. | N.R. | 91.0 | 93.0 | N.R. | N.R. | N.R. | N.R. |
| 83.4 | 37.5 | N.R. | N.R. | N.R. | N.R. | N.R. | N.R. | 88.0 | 93.0 | N.R. | N.R. | N.R. | N.R. |
| 4.9 | 4.2 | N.R. | N.R. | N.R. | N.R. | N.R. | N.R. | N.R. | N.R. | N.R. | N.R. | N.R. | N.R. |
| N.R. | N.R. | 11.3 | 18.0 | 78.5 | 87.3 | N.R. | N.R. | 83.6 | 96.3 | N.R. | N.R. | 14.7 | 8.1 |
| 2.7 | 2.4 | N.R. | N.R. | N.R. | N.R. | 62.9 | 7.3 | N.R. | N.R. | 36.3 | 3.3 | 11.9 | 6.6 |
| 2.4 | 2.1 | N.R. | N.R. | N.R. | N.R. | N.R. | N.R. | N.R. | N.R. | N.R. | N.R. | N.R. | N.R. |
| N.R. | N.R. | N.R. | N.R. | N.R. | N.R. | N.R. | N.R. | N.R. | N.R. | N.R. | N.R. | N.R. | N.R. |
| N.R. | N.R. | N.R. | N.R. | N.R. | N.R. | N.R. | N.R. | N.R. | N.R. | N.R. | N.R. | N.R. | N.R. |
| 2.9 | 3.0 | N.R. | N.R. | N.R. | N.R. | N.R. | N.R. | N.R. | N.R. | N.R. | N.R. | N.R. | N.R. |
| 3.6 | 3.0 | N.R. | N.R. | N.R. | N.R. | N.R. | N.R. | N.R. | N.R. | N.R. | N.R. | N.R. | N.R. |
| N.R. | N.R. | 19.5 | 21.6 | 87 | 86 | N.R. | N.R. | N.R. | N.R. | N.R. | N.R. | 6.5 | 4.1 |
| 89.6 | 88.9 | N.R. | N.R. | N.R. | N.R. | N.R. | N.R. | N.R. | N.R. | N.R. | N.R. | 13.2 | N.R. |
| 4.4 | 0.8 | N.R. | N.R. | N.R. | N.R. | N.R. | N.R. | N.R. | N.R. | N.R. | N.R. | 8.0 | N.R. |
| 9.4 | 0.8 | N.R. | N.R. | N.R. | N.R. | N.R. | N.R. | N.R. | N.R. | N.R. | N.R. | 7.1 | N.R. |
| 1.7 | 1.6 | N.R. | N.R. | N.R. | N.R. | N.R. | N.R. | 98.5 | 98.5 | N.R. | N.R. | N.R. | N.R. |
| 2.2 | 2.0 | N.R. | N.R. | N.R. | N.R. | N.R. | N.R. | N.R. | N.R. | N.R. | N.R. | N.R. | N.R. |
| N.R. | N.R. | N.R. | N.R. | N.R. | N.R. | 24.0 | 3.0 | N.R. | N.R. | 2.2 | 0.0 | 8.0 | 3.0 |
| 6.4 | 7.2 | N.R. | N.R. | N.R. | N.R. | N.R. | N.R. | N.R. | N.R. | N.R. | N.R. | 7.8 | 6.4 |
| 3.5 | 3.4 | N.R. | N.R. | N.R. | N.R. | 1.2 | 0.3 | N.R. | N.R. | N.R. | N.R. | N.R. | N.R. |
| 0.7 | 0.6 | 16.2 | 17.2 | 81 | 88 | N.R. | N.R. | 87.0 | 93.0 | 0.1 | 0.0 | 9.0 | 8.0 |
| N.R. | N.R. | N.R. | N.R. | N.R. | N.R. | 5.6 | 1.0 | N.R. | N.R. | N.R. | N.R. | 7.0 | 7.0 |
| N.R. | N.R. | N.R. | N.R. | N.R. | N.R. | 15.0 | 1.0 | N.R. | N.R. | N.R. | N.R. | 7.3 | 7.0 |
| N.R. | N.R. | 17.8 | 19.9 | 79.4 | 86.5 | 5.9 | 1.0 | N.R. | N.R. | N.R. | N.R. | N.R. | N.R. |
| N.R. | N.R. | 17.0 | 19.9 | 84.6 | 86.5 | 18.3 | 1.0 | N.R. | N.R. | N.R. | N.R. | N.R. | N.R. |
| N.R. | N.R. | N.R. | N.R. | N.R. | N.R. | N.R. | N.R. | N.R. | N.R. | N.R. | N.R. | N.R. | N.R. |
| 11.0 | 11.1 | N.R. | N.R. | N.R. | N.R. | 1.5 | 0.6 | 95.5 | 96.4 | 47.0 | 6.0 | N.R. | N.R. |
| 1.9 | 1.5 | N.R. | N.R. | N.R. | N.R. | N.R. | N.R. | 91.9 | 94.6 | 4.8 | 0.0 | 10.0 | 6.7 |
| 3.3 | 1.5 | N.R. | N.R. | N.R. | N.R. | N.R. | N.R. | 89.3 | 94.6 | 29.2 | 0.0 | 11.3 | 6.7 |
| N.R. | N.R. | N.R. | N.R. | N.R. | N.R. | N.R. | N.R. | N.R. | N.R. | N.R. | N.R. | N.R. | N.R. |
| 3.9 | 2.6 | N.R. | N.R. | N.R. | N.R. | 16.9 | 1.6 | N.R. | N.R. | N.R. | N.R. | 7.8 | 5.8 |
| N.R. | N.R. | 12.0 | 26.4 | 81.6 | 89.6 | N.R. | N.R. | 84.7 | 90.7 | 39.4 | 2.5 | N.R. | N.R. |
| N.R. | N.R. | N.R. | N.R. | N.R. | N.R. | 23.7 | 5.7 | 87.4 | 91.4 | 15.0 | 0.1 | N.R. | N.R. |
| 0.6 | 0.3 | N.R. | N.R. | N.R. | N.R. | N.R. | N.R. | N.R. | N.R. | N.R. | N.R. | N.R. | N.R. |
| N.R. | N.R. | 13.2 | 19.2 | 75.6 | 90.2 | 56.5 | 1.4 | 72.9 | 93.7 | N.R. | N.R. | N.R. | N.R. |
| N.R. | N.R. | N.R. | N.R. | N.R. | N.R. | N.R. | N.R. | N.R. | N.R. | N.R. | N.R. | N.R. | N.R. |

Abbreviations: OSAS, obstructive sleep apnea syndrome; W/O Hypert-DM, without hypertension and diabetes mellitus; SBP, systolic blood pressure; DBP, diastolic blood pressure; TC, total cholesterol; HDLC, high-density lipoprotein cholesterol; LDLC, low-density lipoprotein cholesterol; CRP, C-reaction protein; IL-6, interleukin 6; REM, rapid eye movement; ODI, oxygen desaturation index; SaO2, arterial xyhemoglobin saturation; ESS, Epworth sleepiness scale.
